# Larval descriptions of three *Dromica* species with some bionomical remarks (Coleoptera, Cicindelidae)

**DOI:** 10.3897/zookeys.1044.61993

**Published:** 2021-06-16

**Authors:** Peter Schüle, Alexander Putchkov, Tetiana Markina

**Affiliations:** 1 Rosenstrasse 9, 71083, Herrenberg, Germany Unaffiliated Herrenberg Germany; 2 Institute of Zoology, National Academy of Sciences of Ukraine, vul. B. Khmelnytskogo, 15, Kyiv-30, MSP, 01030, Ukraine Institute of Zoology, National Academy of Sciences of Ukraine Kyiv Ukraine; 3 H.S. Skovoroda Kharkiv National Pedagogical University 61002, Alchevskykh str., 29, Ukraine H.S. Skovoroda Kharkiv National Pedagogical University Kharkiv Ukraine

**Keywords:** Diagnostic remarks, ecology, morphology, South Africa, Tiger beetle larvae

## Abstract

Larvae of all instars of Dromica (s. str.) kolbei (W. Horn, 1897), D. (s. str.) alboclavata Dokhtouroff, 1883 and instar I of D. (s. str.) helleri (W. Horn, 1897) are described and figured in detail. The larvae of these species have several unique morphological characters. The shape of the pronotum, appendages of abdominal segment V and the peculiarities of chaetotaxy are of taxonomic importance. The main diagnostic characters to separate these species from each other and from other known *Dromica* larvae are given and discussed. Some bionomical peculiarities of D. (s. str.) kolbei are presented.

## Introduction

The genus *Dromica* was established as a monotypic genus for the species *Dromica coarctata* (Dejean, 1826) originally described as *Cicindela coarctata* Dejean in Latreille and Dejean, 1822. The two genera *Myrmecoptera* Germar, 1843 and *Cosmema* Boheman, 1848, initially described as distinct genera, have subsequently been regarded as synonyms of the genus *Dromica* (Horn 1935, 1940; Wiesner 1992; Werner 1999; Cassola 2002; Lorenz 2005). Cassola (2002) separated two new genera *Pseudodromica* and *Foveodromica* from the genus *Dromica* by using morphological characters (body size, form of pronotum, narrow labial palpi, and the shape of aedeagus). Moreover, Cassola divided *Dromica* s. str. into nine species groups. However, the morphological characters used by Cassola were ambiguous and *Pseudodromica* and *Foveodromica* are currently regarded as subgenera of the genus *Dromica* by some authors (e.g., Lorenz 2005; Anichtchenko 2014; Serrano et al. 2017; Schüle and Monfort 2018; Putchkov et al. 2018, Wiesner 2020). All species of *Dromica* (almost 170) are African endemics, and most of them occur in southern Africa (especially in Republic of South Africa).

In contrast to adults, information about the larvae is very scarce and larval stages of only two species have been described until now (Arndt 1998; Putchkov et al. 2018). To compensate for the lack of data on the larval morphology and to provide background for understanding the changing history of the generic nomenclature, one of us (P. Schüle) decided to rear various *Dromica* species for comparative studies. The information gathered on the larval morphology of two species of the subgenus Pseudodromica was presented by Putchkov et al. (2018).

This work comprises the descriptions of three species belonging to two different “species groups” of the subgenus Dromica (s. str.): *D. alboclavata* Dokhtouroff, 1883 (group “*coarctata* / *furcata* / *marginella*”), *D. kolbei* (W. Horn, 1897), and *D. helleri* (W. Horn, 1897) (group “*sexmaculata*”).

## Material and methods

The descriptions are based on the following larval material:

*Dromica kolbei* (3 LI, 7 LII, 6 LIII), South Africa, RSA, Northern Province, Ben Lavin Nature Reserve near Louis Trichardt, open bushfield area, 29 Jan 2000, leg. P. Schüle;

*Dromica helleri* (1 LI), South Africa, Eswatini [Swaziland], Mlawula NR, 22 Nov 2001, leg. P. Schüle;

*D. alboclavata* (3 LI; 3 LII; 1 LIII), South Africa, RSA, Gauteng, Hartbeestpoort, 5 Dec 2004, leg. P. Schüle.

The larvae were reared ex ovo in laboratory conditions. The nomenclature follows Lorenz (2005) and partly Cassola (2002). Terminology of morphology and chaetotaxy of larvae follows Arndt (1998), Hamilton (1925), Knisley and Pearson (1984), Putchkov (2013), Putchkov and Arndt (1994), and Putchkov and Cassola (1994). The morphological terms are abbreviated as follows:

**HL** head length (from nasale apex to the end of fronto-clypeal-labral area);

**HW** head width measured at its broadest portion, usually at the level of stemmata I–II;

**PNL** pronotum length measured along midline;

**PNW** pronotum width measured at its broadest portion, usually at level of cephalolateral angles or slightly below;

**A1, A2** first and second antennal segments;

**LP1** first labial palpus;

**LP2** second labial palpus;

**ST1**, **ST2** first and second stemmata;

**PN** pronotum;

**PN1** half of pronotum with number of discal setae (but without marginal setae);

**PNa** cephalolateral (anterior) angles of pronotum;

**PNm** median line of pronotum;

**T3** third abdominal tergite;

**HY** hypopleuron,

**AT5** apical tergite;

**CT5** caudal tergite;

**CTL5** lateral tergite of abdominal segment V (hump);

**MH** medial hook;

**IH** inner hook on abdominal segment V;

**EU9** posterior part of abdominal sternite IX;

**TE9** posterior part of abdominal tergite IX;

**PY** pygopod.

Projections of MH and IH of abdominal segment V are figured in dorsal and partly in lateral views. Length of hooks are measured from the posterior margin at base to the apex of hooks (diagonally). The sizes are given in mm. All larval material is deposited in the entomological collections of Schmalhausen Institute of Zoology NAS of Ukraine (**SIZK**).

## Results

### Dromica (s. str.) kolbei

Taxon classificationAnimaliaColeopteraCicindelidae

(W. Horn, 1897)

C80FC2C9-1F55-589F-949D-617A48B54DBD

#### Instar III.

**Figure 1.**
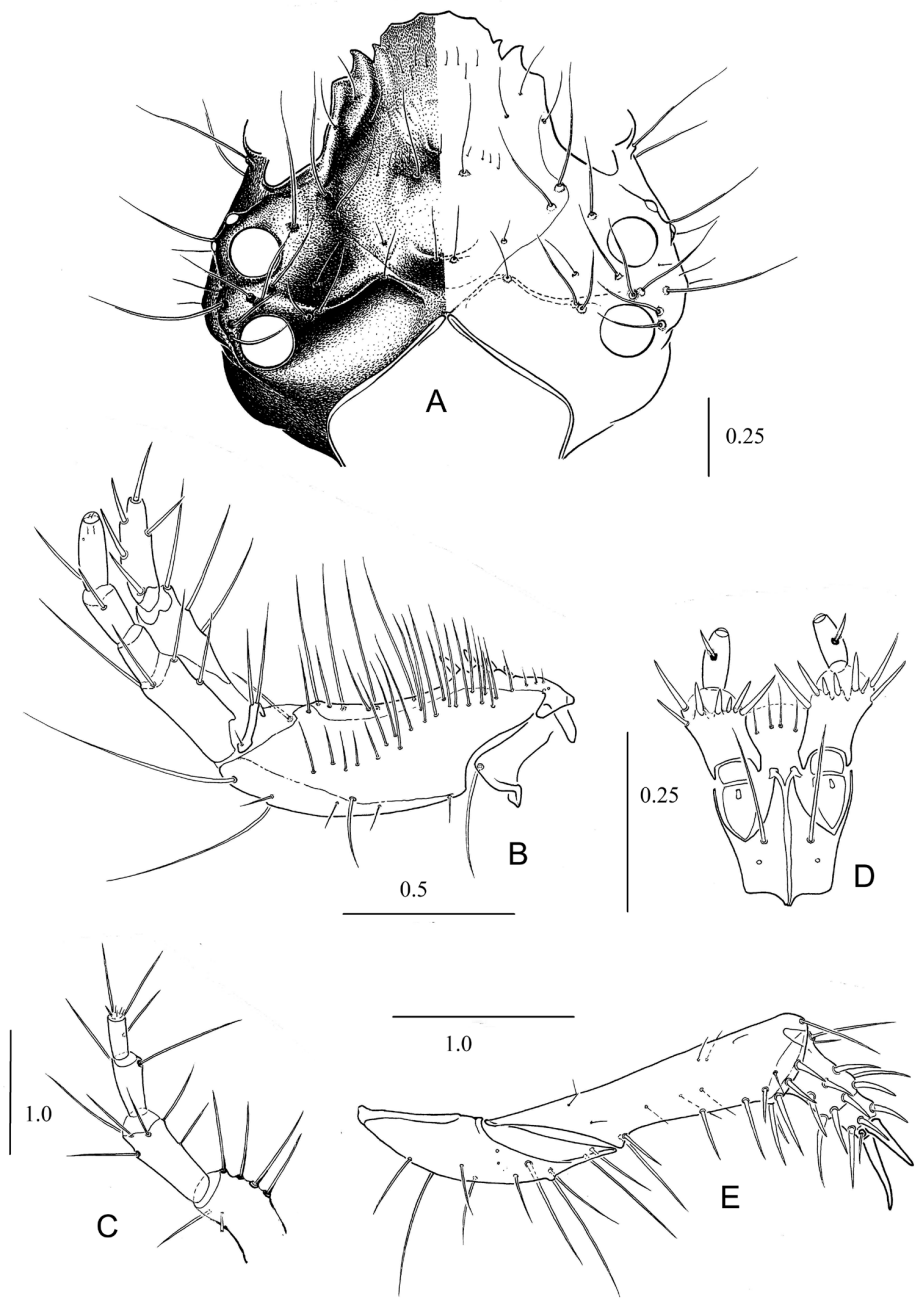
Head and appendages of *Dromica kolbei*, III instar **A** head (above) **B** left maxilla (above) **C** left antenna (above) **D** labium (below) **E** first right leg (below).

**Figure 2.**
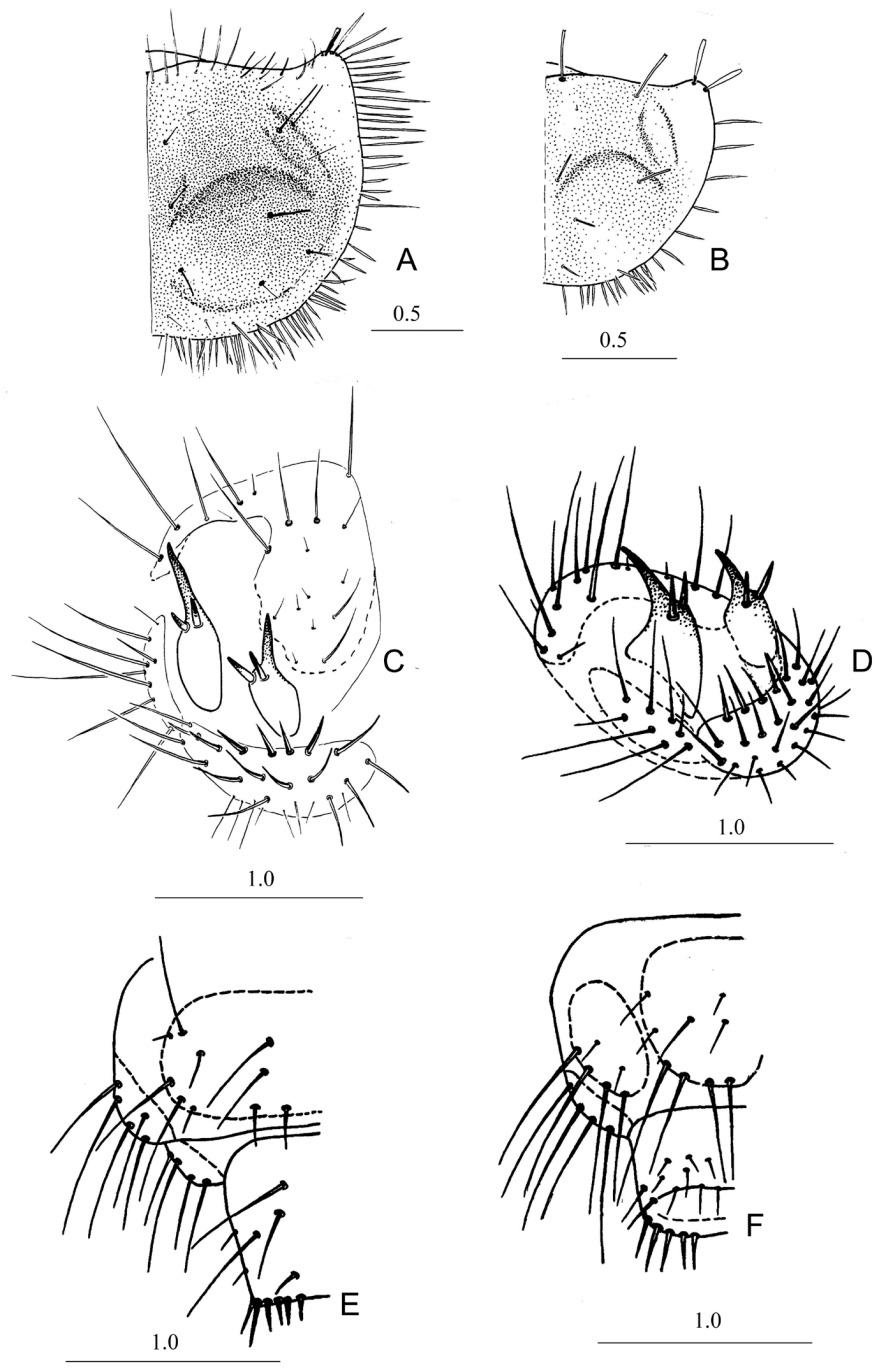
Morphological features of *Dromica kolbei* larvae **A** pronotum, III instar (right half, above) **B** pronotum, I instar (right half, above) **C** hump, III instar (left part, dorsal view) **D** the same (lateral view) **E** segment IX and pygopod of abdomen, III instar (dorsal view) **F** segment IX and pygopod of abdomen, III instar (ventral view).

*Measurements*: FL 1.70–1.90 (1.80); FW 2.80–3.15 (3.01); PNL 2.05–2.25 (2.14); PNW 3.40–3.75 (3.59). *Head*. Disk of head posteriorly light brown, almost yellow on anterior third. Remainder of head dark brown with bright greenish blue luster. Ventral side of head dark brown. Most setae of head dark brown or almost black, not flattened. Only some setae on anterior part of clypeus brown or light brown. Mandibles and stipes of maxillae dark brown. Galea light brown. A1 and A2 yellow (or slightly reddish), A3 and A4 brown. Adnasalia distinct and not shorter than lateral teeth (Fig. 1A). Apexes of adnasalia slightly curved outside. Lateral plates (below adnasalia) shortened, its apexes almost rectangular. Tubercles between ST1 and ST2 distinct with two setae. Width of A1 1.2–1.3 × wider than A2 and 3–4 × wider than A3 and A4. Anterior lateral margin of A1 and A2 slightly flattened. A1 with six setae; A2 with five setae in upper part; A3 with two or three setae; A4 with two or three long and six or seven very short setae (Fig. 1C). Maxillae show the typical form for Cicindelini larvae (Fig. 1B). Galea slightly shorter than maxillary palpus. LP1 with seven or eight spinelets (two or three marginal ones slightly longer). LP2 with one seta almost in the middle (Fig. 1D). Epicranial suture absent. *Thorax*. PN brown with light brown anterior margin. PNa light yellow, wide, rounded and directed forwards. Width of PN in 1.66–1.68 × wider than long, its maximum width in the middle. Callous elevations of PN and swellings of PNa distinct. Anterior margin of PN slightly concave in the middle and as long as or slightly shorter than tips of PNa (Fig. 2A). Setae of anterior margin and disk of PN dark brown, but lateral and posterior setae yellow or pale. Most setae long, thin and acuminate (only one or two setae on tips of PNa slightly flattened). Anterior margin of PN with 12–14 setae; PN1 with 11–13 anterior setae (without marginal setae) and swellings of PNa with two setae. First pair of legs dark brown, other pairs yellow brown. Legs typical for Cicindelina larvae (Fig. 1E). *Abdomen*. Sclerotized areas of abdomen indistinct, pale yellow. T3 with 8–10 setae. HY consisting of one large posterior and one or two small anterior sclerites. Inner margins of tergites of hump almost contacting. CT5 and CTL5 fused. CTL5 with 7–9 long setae. CT5 transverse-elongated with 15–18 stout bristles and some small setae (Fig. 2C, D). Hooks of hump widened basally, their tops moderately curved downwards (in lateral view) (Fig. 2D). Length of MH 1.7–1.8 × longer than width at base. Apex of MH reach middle of AT5. Length of IH 1.25–1.30 × shorter than MH. Central spine of IH with distinctly longer lateral bristles. Spines of MH and IH are almost symmetrical and situated slightly in front of middle (Fig. 2C, D). EU9 with eight long setae (Fig. 2F). PY dorsally with eight or nine long and two or three shorter setae, ventrally with 10–12 small setae (Fig. 2E, F). Top of PY with 16 or 17 dorsally stout setae.

#### Instar II.

*Measurements*: FL 1.17–1.19; FW 2.00–2.20; PNL 1.35–1.40; PNW 2.05–2.08. Differing from instar III mainly by number of setae on some appendages and sclerites. *Head*. Coloration of head and pronotum same as in instar III but less bright. Most setae on head transparently white and only brown near eyes; A1 with five setae (three on anterior lateral margin). A2 apically with five long setae; A3 with two long, and A4 with two or three long and 4–6 small setae. Galea slightly shorter than maxillary palpus. LP1 with six spinelets, LP2 with one seta slightly before the middle. Epicranial suture absent.

*Thorax.*PN 1.48–1.52 × wider than long. Anterior margin of PN almost even or slightly concave. Most setae of PN1 slightly flattened and truncated (in instar III: thin and acuminate). Anterior margin of PN with 24–27, PN1 with 8–11 and swellings of PNa with one or two setae. *Abdomen* Sclerotized areas of abdomen indistinct. T3 with 8–10 setae, HY consisting of one large posterior and one small anterior sclerite. CTL5 with four long setae. Anterior part of CT5 with 12–16 stout bristles and some thin setae. Central spine of IH longer than lateral bristles. EU9 with six long setae. PY with 8–10 setae dorsally and six or seven setae ventrally. Apex of PY with 16 setae (8–10 of them stouter).

#### Instar I.

**Figure 3.**
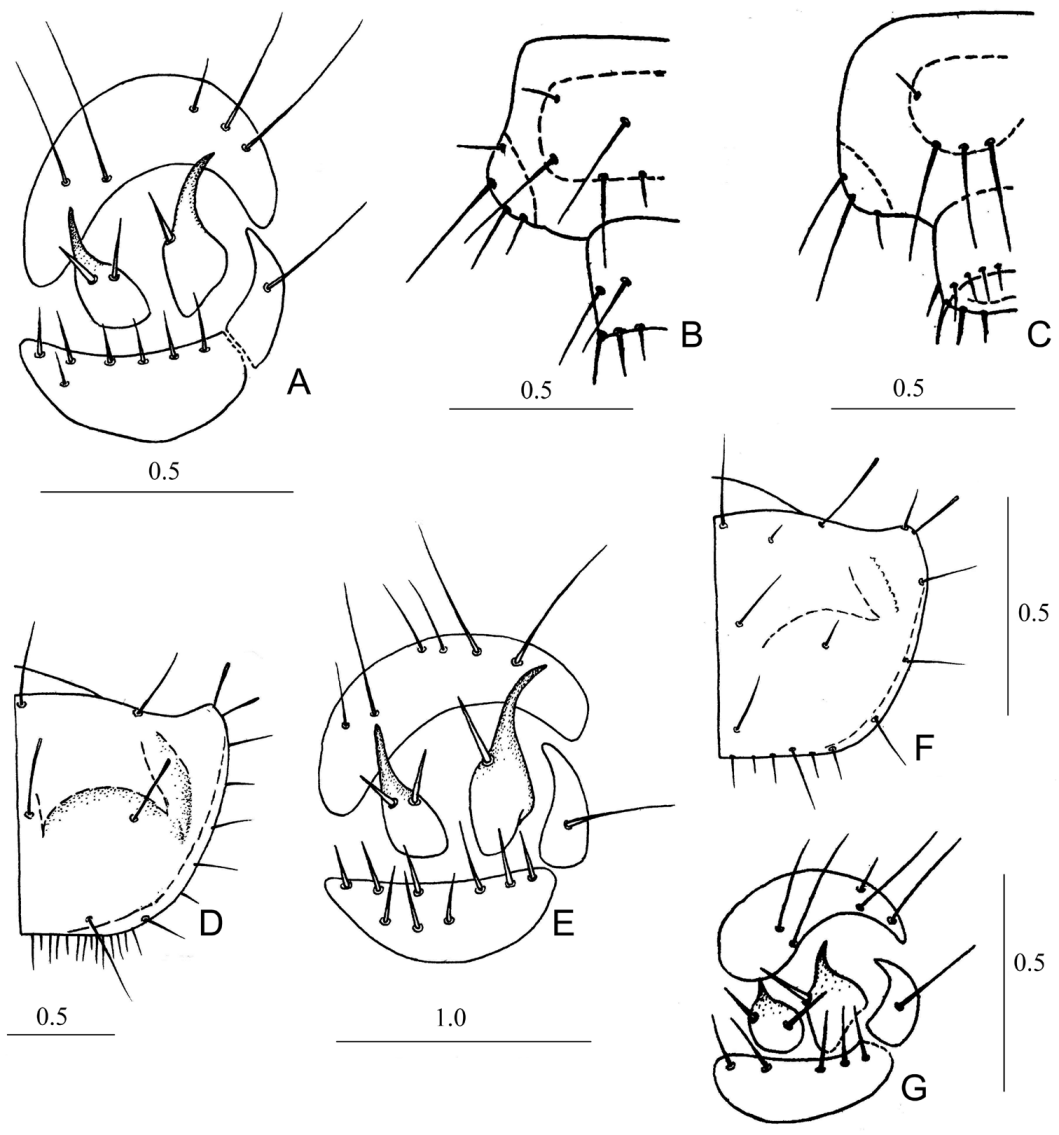
Morphological features of *Dromica* larvae, I instar **A** hump of *D. kolbei* (right part, dorsal view) **B** segment IX and pygopod of abdomen of *D. kolbei* (left half, dorsal view) **C** segment IX and pygopod of abdomen of *D. kolbei* (left half, ventral view) **D** pronotum of *D. helleri* (right half, above) **E** hump of *D. helleri* (right part, dorsal view) **F** pronotum of *D. alboclavata* (right half, above) **G** hump of *D. alboclavata*.

*Measurements*: FL 0.8; FW 1.35; PNL 0.95; PNW 1.50. *Head.* Anterior third of head light yellow, clypeus brown, lateral margin of head (near eyes) dark brown with greenish metallic luster. All setae of head transparently reddish, slightly flattened and truncated. Apexes of lateral plates near adnasalia shortened, slightly rounded. Tubercles between ST1 and ST2 with one seta. A1 glabrous; A2 and A3 with two long setae; A4 with three long and several small setae on apex. Galea shorter than maxillary palpus. LP1 with three spinelets; LP2 with one seta before middle. *Thorax*. PN light brown, PNA almost yellow. PN 1.58 × wider than long. Tips of discal setae of PN slightly obtuse. Maximum width of PN in the middle. Anterior margin of PN almost even, longer than widened and rounded tops of PNa (Fig. 2B). Median setae of anterior margin and disk of PN dark brown, thin, and obtuse. Setae on tips of PNa white and flattened. PN1 with five setae (Fig. 2B). *Abdomen*. Sclerotized areas of abdomen reddish, more distinct than in instars II and III. T3 with three or four, CTL5 with one, CT5 with six or seven setae. MH with one stout bristle in the middle. Two bristles on IH placed almost symmetrically (Fig. 3A). EU9 with six setae (Fig. 3C). Apex of PY with 12 or 13 setae (dorsally six setae stouter) (Fig. 3B, C).

#### Distribution and ecology.

*Dromica kolbei* is distributed in northern South Africa and in southern Zimbabwe. The single record from Angola still needs to be confirmed (Cassola 2002). This species inhabits dry savanna areas with scattered trees and bushes and open forests on sandy soil.

#### Bionomical peculiarities.

In early February 2000, two adults of *D. kolbei* were brought back alive to the laboratory in Germany, where they were placed in an open bowl (40 cm × 50 cm × 14 cm) filled with 8 cm of loamy sand, and lit by a 12 V 50 W light bulb 12 hours per day.

The first copulation could be observed during the second day and took only a few minutes, just as all the subsequent copulations. After a few days, the female refused further mating activities by the male. Even if the male’s mandibles successfully gripped the female between the thorax and the elytra (the coupling sulcus being marked by a longitudinal shallow impression on the upper half of the mesepisternum), the male did not succeed in inserting its fully protruding aedeagus. By arrhythmic waggling of the abdomen, the female tried to get rid of the male. Zikan (1929) observed a similar behavior in other genera of Cicindelidae.

While mating, the male spread out the prolegs, tightly holding the female with its mandibles. The male’s labial palpi, with second palpomeres inflated, flattened and with rows of long setae on the upper surface, were folded backwards and pressed onto the female’s elytra. The use of the male’s dilated protarsi, bearing brush-like adhesive hairs, could not be observed.

Egg laying only took place immediately after watering the substrate. Initially, the female tested the substrate by touching the surface with its palpi, lowering its head while walking slowly. After the female made a small hole by digging with its abdomen, most probably by using its cerci as tools. The abdomen was placed into this hole up to the penultimate tergite, while the ovipositor was extended for ca. 8–10 mm for egg laying.

The first eggs were laid on 6 March. The egg-laying period lasted until the female’s death on 9 June 2000 (95 days later). During this period, the female dug ca. 150 holes. When the female was disturbed by the male, she stopped laying eggs and moved to another area; therefore many holes did not contain any eggs. Furthermore, no larvae left the burrows that had been dug at the end of the egg-laying period.

After hatching, the coiled larvae remained in a small cavity for a few days before digging a circular tunnel to the substrate surface. During the 1^st^ larval instar, the opening of this tunnel was simply rounded. In front of the oblique tunnel opening, the 2^nd^ and 3^rd^ instar dug a transverse cavity, which was larger for the 3^rd^ instar. Sometimes, the tunnel opening was located at the hollowed lateral wall instead of its top. Approximately seventy larvae hatched from eggs and ca. 40 larvae reached the 3^rd^ larval instar (fifteen larvae were killed and added to the second author´s collection). The first larvae could be observed 33 days after the beginning of the egg-laying period. The shortest period between the first instar I larva and the appearance of the instar II larva numbered 21 days while there were 49 days between the first appearance of an instar II larva and the appearance of the instar III larva. After several months, all the instar sizes could be found. In January 2001, seven months after the last eggs had been laid, one specimen of instar I appeared at the surface. In January 2003, the last living instar I larva of the first generation was dug out, 31 months after the last eggs had been laid.

The first pupa was discovered at the beginning of August 2001. The position of the pupa was vertical in a simple cave ca. 1 cm underneath the surface. The first imago of the first generation appeared in September 2001. Several pupae were dug up and placed in a humid box. The hatching of one male was observed: it took ca. 10 hours from the beginning until the elytra were fully developed and another two days for the full coloration to develop. This specimen lived from 31 August to 28 October 2001.

One female was dug up fully developed on the 31 of August. This female started with egg laying 7 weeks later, on 19 October but died on 2 February, after having lived for 5 months. Two eggs with fully developed larvae inside and belonging to the F2 generation were dug up on 6 February. The F2 generation egg mortality was very high, although dozens of eggs were laid, not a single larva hatched.

### Dromica (s. str.) helleri

Taxon classificationAnimaliaColeopteraCicindelidae

(W. Horn, 1897)

65520444-8A9F-587A-AA70-5F0F41440171

#### Instar I.

*Measurements*: FL – 0.85; FW – 1.48; PNL – 0.95; PNW – 1.58. *Head*. Head and appendages light brown (only A3 and A4 are darkened). Most setae on clypeus transparently white with reddish hue, setae of occiput (especially near eyes) dark brown. Lateral part of head (near eyes) brown with slight metallic luster. Most setae of head thin and acuminate, (only some setae apically slightly flattened). Outer margin of lateral plates of clypeus slightly curved. A1 glabrous, A2 and A3 with two setae, A4 with two long and several short setae on apex. Galea distinctly shorter than maxillary palpus. LP1 with three bristles; LP2 with one seta before middle. Epicranial suture absent.

*Thorax*. Disk of PN brown, PNa light yellow. Setae of PN1 dark brown (but lateral and posterior setae transparent white). PN 1.66 × wider than long. Anterior margin of PN almost even, slightly longer than small apices of PNa (Fig. 3D). Maximum width of PN on level of PNa. Tops of PNa directed forwards. Callous elevations of PN1 and swellings of PNa distinct. PN1 with four setae. Anterior pair of legs brown, other light brown. *Abdomen*. Sclerotized areas of abdomen indistinct, light reddish. T3 with 3, AT5 with 5–7, CTL5 with one and CT5 with nine setae (3E). MH curved moderately. Their tops reaching middle of AT5 (Fig. 3E). Lateral bristles of IH not in a straight line but slightly displaced asymmetrically. PY with four setae dorsally. Apex of PY with ten setae (four of them stouter on dorsal side).

#### Distribution and ecology.

*Dromica helleri* is recorded from the Republic of South Africa, Mozambique, and Eswatini [Swaziland] (Cassola 2002). In Eswatini, the first author found this species in the low field savanna area of the Siphiso Valley nearby the Mlawula river (riverine floodplain) in an open forest on alluvial soil. The larva of *Dromica helleri* were hatched ex ovo.

### Dromica (s. str.) alboclavata

Taxon classificationAnimaliaColeopteraCicindelidae

Dokhtouroff, 1883

0B251674-C042-5ECB-B011-1B7D4562C7CE

#### Instar III.

**Figure 4.**
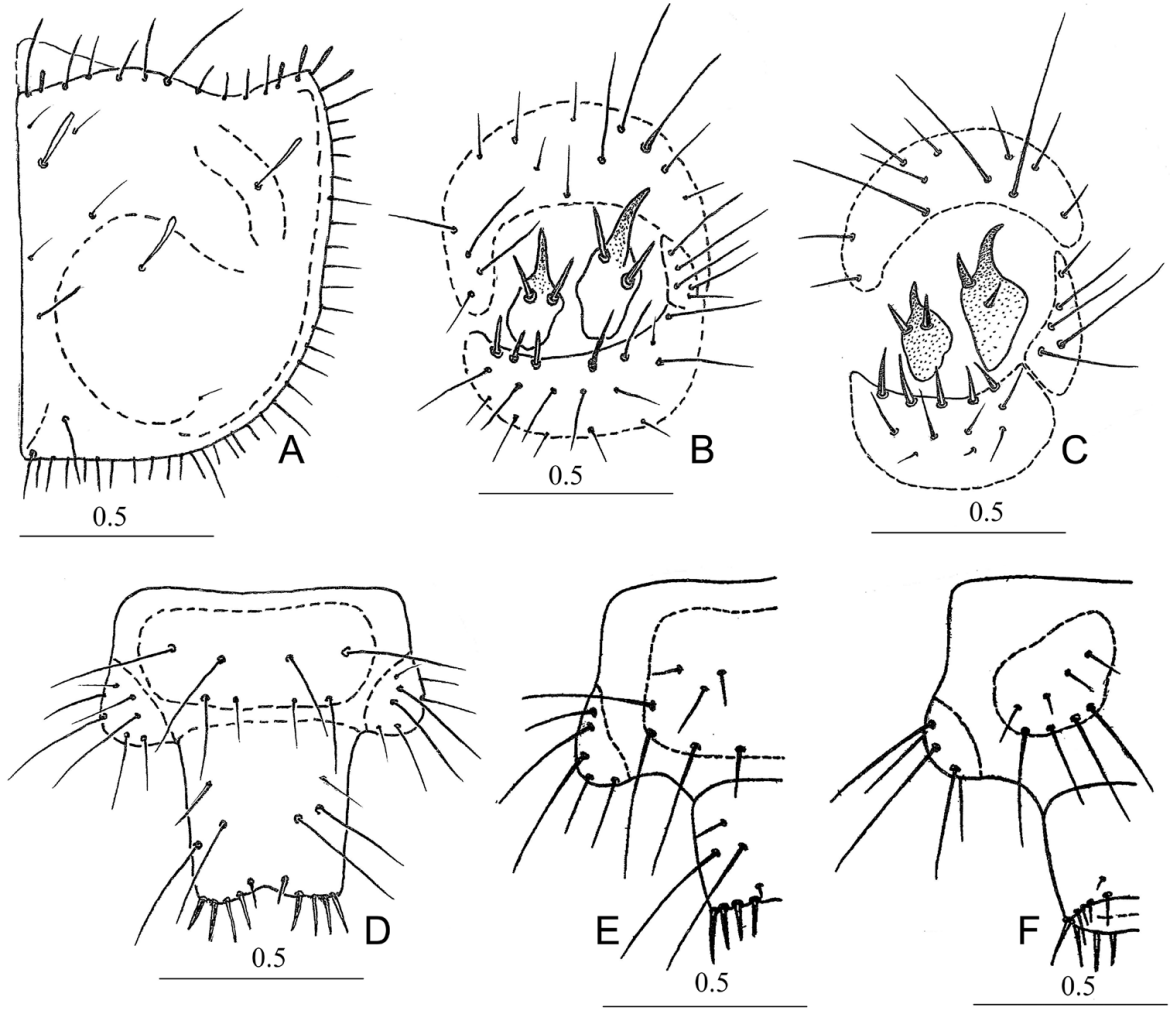
Morphological features of *Dromica alboclavata* larvae **A** pronotum, III instar (right half, above) **B** hump, III instar (right part, dorsal view) **C** hump, II instar (right part, dorsal view) **D** segment IX and pygopod of abdomen, III instar (dorsal view) **E** segment IX and pygopod of abdomen, II instar (left half, dorsal view) **F** segment IX and pygopod of abdomen, II instar (left half, ventral view).

*Measurements*: FL 1.30; FW 2.25; PNL 1.48; PNW 2.58. *Head*. Dark brown above, sometimes almost black, with greenish copper metallic luster. Ventral side of head light brown medially, lateral portions brown. Mandibles dark brown (more darkened apically). Other appendages of head light brown except A3-4 darkened dark brown. Setae of head’s capsule and A1 transparently white, on A2–4 dark brown. Setae of maxillae and labium light brown. Most setae thin and acuminate, only a few setae near ST1 slightly flattened and truncated apically. Lateral plates of clypeus short, almost rectangular. Tubercles between ST1 and ST2 small, but distinct. Lateral portions of head (below eyes) without distinct tubercles. Maximum width of head on level ST1. Antennae relatively large; A1 1.30 time wider than A2 and 2.0–2.6 × wider than A3, A4. A1 slightly longer than A2 and almost 2 × wider than A3 and A4. A1 slightly flattened dorsoventrally on anterior margin and with 6–7 setae (five of them on median part). A2 with five setae on apical part. Ratios of lengths A1:A2:A3:A4 = 1.0:0.9:0.5:0.6. Maxillary palpus distinctly longer than galea. LP2 not shorter than LP1. Seta on LP2 situated slightly before middle. Epicranial suture absent. *Thorax*. PN yellow brown on anterior portions, PNa slightly darkened. Setae transparently white (except 10–13 black setae on anterior margin and near PNa). Callous elevations and swellings of PNa not highly elevated but distinct. Maximum width of PN before middle (Fig. 3F). Anterior margin of PN slightly concave in the middle. PNa widened, its apices almost rounded, directed forwards (Fig. 3F). Ratio PNW/PNL – 1.75. Most setae flattened, some of them truncated apically, only a few setae on anterior margin and near PNa long and acuminate apically. PN1 with 10–12 setae, swellings of PNa with one seta (Fig. 3F). *Abdomen*. Sclerotized areas of abdomen indistinct. T3 with 8–10 setae. HY consisting of one large posterior and one or two small anterior sclerites. AT5 almost fused with CTL5 and connected with CT5 on inner side (Fig. 4A). CT5 with eight or nine stout setae on anterior half and with 6–8 thinner setae medially and on posterior half. MH with two stout setae displaced asymmetrically. Upper half of MH narrower and curved (Fig. 4A). Apex of MH reaching the middle of AT5. MH basally almost 1.20 × wider than long. (Fig. 4A). IH with two almost symmetrical stout setae. Upper part of IH slightly curved. IH 1.3–1.4 × shorter than MH. AT5 with more than ten thin setae, CTL5 with 5 long setae (Fig. 4A). Posterior margin of EU9 with 8 long setae. PY with 8 setae dorsally (two of them shortened). Ventrally PY glabrous. Apex of PY with 16 dorsally stouter setae (Fig. 4D).

#### Instar II.

*Measurements*: FL – 0.93–0.95 (0.94); FW – 1.50–1.60 (1.53); PNL – 1.00–1.05 (1.02); PNW – 1.63–1.75 (1.68). *Head*. Dark brown with bright greenish metallic reflection. Appendages brown or light brown. Setae of head capsule white and transparently white. Setae of A2–4 and other appendages brown or light brown (in A1 sometimes white with brown hue). Nasale long, distinctly trapezoid. Adnasalia small. Lateral teeth relatively wide. Tubercles between ST1 and ST2 distinct. Most setae of head acute but some of them (near eyes) flattened and obtuse on top. A1 with three or four setae on anterior side. A2 with four or five setae on dorsal half. A3 with two, A4 with three long and several short setae. Maxillary palpus slightly longer than galea. LP1 with five or six spinelets (three median ones very short). LP2 with one seta slightly before middle. Epicranial suture distinct but short. *Thorax*. PN light brown without metallic luster. Posterior half of PN light brown. Anterior half and lateral margins of PN light yellow. Most setae of PN white except 10–12 dark brown setae on anterior margin. Tips of PNa with flattened and obtuse setae. Other setae acuminate. Anterior margin of PN slightly concave and longer than PNa. Maximum width of PN in the middle. Callous elevations and swellings of PNa indistinct. PN1 with eight or nine, swellings of PNa with one (rarely two) setae. Legs dorsally light brown, ventrally almost yellow. *Abdomen*. Sclerotized areas of abdomen indistinct. T3 with seven or eight light brown setae. HY consisting of one large posterior and one small anterior sclerite. Tergites of hump not connected. CT5 with seven or eight stout and 4–6 thin setae (Fig. 4B). CTL5 with three to four long setae. MH and IH very widened in basal half but distinctly narrower and bent on apical half (Fig. 4B). MH basally 1.66–1.68 × wider than long. MH with 2 spines placed asymmetrically (one after another) (Fig. 4B). IH 1.65–1.67 × shorter than MH. IH basally 1.22–1.25 × shorter than long. IH with two (rarely three) spines placed almost symmetrically (Fig. 4B). EU9 with eight long setae posteriorly. PY with eight or nine setae dorsally (but apex glabrous) and usually glabrous ventrally. Apex of PY with 15–16 setae on dorsal and lateral areas (eight or nine of them dorsally stouter) (Fig. 4E, F).

#### Instar I.

*Measurements*: FL – 0.57–0.65 (0.60); FW – 0.90–1.04 (0.98); PNL – 0.57–0.65 (0.61); PNW – 0.90–1.07 (0.99). *Head*. Dark brown with greenish luster. Setae transparently white. Lateral teeth of clypeus shorter, lateral margin of adnasalia slightly curved. Setae on clypeus thin and acuminate, setae near eyes distinctly flattened and truncated. Epicranial suture short. *Thorax*. PN light brown or brown with slight metallic luster. PN 1.38 × wider than long. Maximum width of PN shortly before apex of PNa. Anterior margin of PN almost straight in the middle (sometimes slightly concave) and longer than rounded apices of PNa (Fig. 3G). PN1 with four setae. Setae of PN thin, acute and transparently white, except two long, flattened and brown setae on anterior margin. Callous elevations of PN1 and swellings of PNa distinct. *Abdomen*. Sclerotized areas of abdomen indistinct. Tergites of abdominal segment V divided (Fig. 4B). Base of MH and IH very wide but narrow and distinctly curved in upper half. Their apex not reaching posterior margin of AT5. MH with one, IH with two stout lateral setae. Length of central spine of IH shorter (or equal) than lateral setae. CT5 with 3–5, CTL5 with one and AT5 with six setae (Fig. 4B). EU9 and PY slightly darkened dorsally. EU9 with six setae posteriorly. Apex of PY with ten setae (six of them relatively stouter).

#### Distribution and ecology.

*Dromica alboclavata* is a South African species which range is restricted to the Northern parts of the country (Cassola 2002). It lives in dry savanna areas on sandy or loamy-sandy ground. The larvae were hatched ex ovo.

##### Short diagnostic remarks

The larvae of *Dromica* instars II and III can be distinguished from those of other genera of the *Prothymina* complex by the following characters: MH comparatively shortened, basally distinctly widened with curved apices; two short stout spines of MH are situated one after the other; sclerotized areas of abdomen are indistinct or slightly distinct; CT5 with less than 17 spine-like setae (usually 8–13); PNa small and short; PN1 with 9–13 setae; luster on PN indistinct or moderately distinct. All larvae of *Dromica*, described here and in earlier papers (Arndt, 1998; Putchkov et al. 2018; Putchkov, 2020) show some specific morphological features both at subgeneric and specific level. It is possible to separate them by chaetotaxy pattern of MH, IH, PN, coloration of head or pronotum and morphometrically data (sizes, correlation of PNW/PNL, MH/IH). *Dromica tuberculata* (instars II and III) differs from other *Dromica* species by ventrolateral portions of head (underneath the eyes) with a flattened area and a group of 8–10 basally bulbous setae; the anterior margin of PN is almost even, without an impression in front of the small PNa; CT5 usually bears 7–9 bristles; its apices are moderately curved (Putchkov et al. 2018). The antennomere I of *Dromica alboclavata* and *D. clathrata* larvae (instars II and III) is distinctly flattened dorsoventrally. It is at least 1.3–1.4 × wider than antennomere 2; the antennae are more massive than in the other known species; apices of CT5 are distinctly curved and not reaching AT5. *D. kolbei* can be distinguished from these species by relatively thin antennae (Fig. 1C), antennomere I at most 1.2 × wider than antennomere II; CT5 with 12–16 stout setae (Fig. 2C, D, Table 2).

Larvae of instars I of *Dromica* also show some specific morphological features. CT5 of the known larvae of instar I of the subgenus Pseudodromica is glabrous (Putchkov et al. 2018), while those of species of the subgenus Dromica (s. str.) have a row of (3–9) stout setae on anterior margin (Fig. 3A, E, G). First instar of *D. alboclavata* can be distinguished from those of *D. kolbei* and *D. helleri* by distinctly shorter hooks, 3–5 setae on CT5 (Fig. 3G) and smaller size of PN (Table 1). First instars of *D. helleri* can be distinguished from *D. kolbei* by different number of setae on CT5 (Fig. 3A, E), less by size and form of PN (Figs 2B, 3D). In addition, in larvae of different instars there are some differences in the sizes of head and pronotum (Table 1).

Nevertheless, some other characters (form and chaetotaxy of the head, pronotum, hooks, pygopod) are often similar (or overlapping) in all so far known larvae of *Dromica* species (Table 2). Nevertheless the material of the larvae of the genus is very scarce and its comparative analysis presently is still difficult. Currently it is problematically to characterize the taxonomic and phylogenetic significance of the morphological characters of *Dromica* larvae now. For a reliable key the number of described species is still too sparse.

**Table 1.** Measurements of head and pronotum with growth ratio of pronotal width/length of *Dromica* larvae.

Species	Instars	Measurements in mm, mean in parentheses				
		FL	FW	PNL	PNW	PNW/ PNL
*D. clathrata*	I	1.25–1.38 (1.29)	2.05–2.23 (2.14)	1.18–1.23 (1.20)	2.08–2.13 (2.12)	1.73–1.76
	II	1.70	3.20	1.90	3.01	1.58
	III	2.50–2.70 (2.57)	4.00–4.40 (4.20)	2.70–2.90 (2.83)	4.40–4.75 (4.55)	1.62–1.64
*D. tuberculata*	I	1.05	1.80	1.05	1.85	1.76
	II	1.40–1.50	2.43–2.63	1.55–1.73	2.65–2.85	1. 65–1.70
*D. kolbei*	I	0.8	1.35	0.95	1.50	1.58
	II	1.17–1.19	2.00–2.20	1.35–1.40	2.05–2.08	1,48–1,52
	III	1.70–1.90 (1.80)	2.80–3.15 (3.01)	2.05–2.25 (2.14)	3.40–3.75 (3.59)	1.66–1.68
*D. alboclavata*	I	0.57–0.65 (0.60)	0.90–1.04 (0.98)	0.57–0.65 (0.61)	0.90–1.07 (0.99)	1.38
	II	0.93–0.95 (0.94)	1.50–1.60 (1.53)	1.00–1.05 (1.02)	1.63–1.75 (1.68)	1.64–1.67
	III	1.30	2.25	1.48	2.58	1.75
*D. helleri*	I	0.85	1.48	0.95	1.58	1.66

**Table 2.** Chaetotaxy of main segments and sclerites of *Dromica* larvae instar III (abbreviations in Materials and methods).

Species	PN1	PNa	T3	CT5	CTL5	Tip of PY
*D. clathrata*	9–10	2	8–10	8–10	6–8	16–17
*D. kolbei*	11–13	2	8–10	12–16	7–9	16–17
*D. alboclavata*	10–12	1	8–10	7–9	5	16

## Supplementary Material

XML Treatment for Dromica (s. str.) kolbei

XML Treatment for Dromica (s. str.) helleri

XML Treatment for Dromica (s. str.) alboclavata
